# Diagnostic accuracy of tongue coating in identifying acute appendicitis: a prospective cohort study

**DOI:** 10.1136/emermed-2024-214210

**Published:** 2025-04-01

**Authors:** Hideki Mori, Kazumi Yamasaki, Yusuke Saishoji, Yuichi Torisu, Takahiro Mori, Yuki Nagai, Yasumori Izumi

**Affiliations:** 1 Department of General Internal Medicine, NHO Nagasaki Medical Center, Omura, Nagasaki, Japan; 2 Clinical Research Center, NHO Nagasaki Medical Center, Omura, Nagasaki, Japan

**Keywords:** Diagnostic Techniques and Procedures, diagnosis, Diagnostic Tests, gastro-intestinal

## Abstract

**Background:**

Acute appendicitis requires timely diagnosis. The diagnostic efficacy of tongue examination in making this diagnosis has not been established. This study investigates whether the Tongue Coating Index (TCI), a validated measure of tongue coating, can aid in diagnosing acute appendicitis.

**Methods:**

We conducted a prospective cohort study (1 September 2018–31 December 2020) at a single Japanese hospital. Adults (≥20 years) with suspected acute appendicitis, presenting to either the emergency department or general outpatient clinic, were enrolled. Tongue images were taken at presentation; two independent examiners—unrelated to clinical care and blinded to patient data—later scored these images using the TCI. A composite reference standard (clinical findings, imaging, histopathology, follow-up) was used to confirm appendicitis. We compared the TCI’s diagnostic performance with the Alvarado score and its components using C-index, area under the curve (AUC), sensitivity and specificity.

**Results:**

Of 145 included patients, 69 (47.6%) were diagnosed with acute appendicitis. The TCI demonstrated comparable discriminative ability (C-index AUC 0.62; 95% CI, 0.53 to 0.71) to that of the Alvarado score (0.66; 95% CI, 0.57 to 0.75). Of Alvarado score components, migration of pain had an AUC of 0.63 (95% CI, 0.55 to 0.71), anorexia 0.58 (95% CI, 0.50 to 0.66) and tenderness in the right lower quadrant 0.55 (95% CI, 0.50 to 0.60). At a cut-off of 3, the TCI demonstrated high sensitivity of 96% (95% CI, 88% to 98%) but low specificity of 21% (95% CI, 13% to 32%). Conversely, at a cut-off of 10, the TCI showed increased specificity of 83% (95% CI, 73% to 90%) but reduced sensitivity of 29% (95% CI, 20% to 41%).

**Conclusion:**

The TCI showed comparable diagnostic performance to the Alvarado score and its individual components. TCI may potentially serve as an additional non-invasive indicator for diagnosing or ruling out acute appendicitis. Further research is essential to validate its clinical utility.

**WHAT IS ALREADY KNOWN ON THIS TOPIC**

- The Alvarado score is widely used to diagnose acute appendicitis, but its accuracy is moderate and it relies on blood tests. In particular, in settings where laboratory or imaging studies are not readily available, a non-invasive diagnostic approach is valuable. Tongue coating has been reported as a simple indicator of intra-abdominal inflammation. Therefore, if proven useful for diagnosing acute appendicitis, it could serve as a rapid diagnostic adjunct.

**WHAT THIS STUDY ADDS**

- In this prospective study of adults with suspected appendicitis, the Tongue Coating Index (TCI) demonstrated diagnostic performance comparable to the Alvarado score, suggesting that the degree of tongue coating may assist in clinical decision-making. While further validation is required, the TCI could offer a rapid, accessible diagnostic adjunct.

**HOW THIS STUDY MIGHT AFFECT RESEARCH, PRACTICE, OR POLICY**

- TCI could be integrated into diagnostic protocols and might be most useful in settings where traditional diagnostic methods are less accessible, potentially informing decisions about further investigations.

## Introduction

 Acute appendicitis is one of the most common abdominal emergencies,^1^ with delayed treatment often resulting in serious complications.^2 3^ Therefore, early and accurate diagnosis is crucial. However, diagnosing appendicitis based solely on symptoms or physical findings remains challenging.^4^ Blood tests and imaging modalities, such as abdominal CT and ultrasonography (US), play a significant role in diagnosis,^5^ it is important to optimise their use due to concerns regarding radiation exposure, cost and time. Furthermore, these tools may not easily be available in low-resource settings^6^ and require additional expense and often referrals to higher-level hospitals. In addition, consequently, a diagnostic strategy that may help in early evaluation may provide guidance for further investigation.

Several clinical prediction rules have been developed to aid in diagnosing acute appendicitis.^7^ The Alvarado score, published in 1986, is a widely used tool that combines clinical findings and blood tests.^8^ While useful, the score’s reliance on blood tests reduces its practicality for rapid diagnosis in situations where blood sampling is unavailable. Moreover, its overall diagnostic accuracy has been reported as moderate, underscoring the need for more efficient diagnostic tools.^9 10^

The use of tongue coating as a diagnostic feature for acute appendicitis was initially described over 70 years ago.^11^ Tongue examination offers several advantages: it is simple, non-invasive, requires no specialised equipment and places minimal burden on the patient. These factors make tongue inspection a potentially valuable tool for diagnosing appendicitis, particularly in low-resource environments.

In this work, we aimed to evaluate the diagnostic utility of tongue examination in acute appendicitis by examining the performance of the Tongue Coating Index (TCI), a previously validated measure for assessing tongue coating.^12^

## Methods

### Ethical considerations

This is a prospective cohort study that is reported in accordance with the Standards for Reporting of Diagnostic Accuracy Studies guidelines.^13^

This study adheres to the Declaration of Helsinki. Before enrolment, each patient was informed about the study through written documentation and provided written informed consent.

### Data source and participants

This study was conducted at the National Hospital Organization Nagasaki Medical Center, a tertiary medical institution in Nagasaki, Japan, and included adult patients aged≥20 years who visited the emergency department or outpatient clinic of the institution between 1 September 2018 and 31 December 2020 with suspected appendicitis. In the Japanese healthcare system, general outpatient clinics in acute care hospitals often accommodate patients with acute conditions, such as suspected appendicitis. Suspected appendicitis was determined by the attending physicians based on the patients’ symptoms and physical examination findings. Patients who had previously undergone an appendectomy, those incapable of making decisions due to dementia or mental illness, individuals unable to follow instructions or deemed uncooperative and cases where tongue observation was difficult due to underlying conditions were excluded. Enrolment followed a consecutive sampling approach. Diagnostic imaging studies, such as CT, US and MRI were performed based on the treating physician’s clinical judgement. Furthermore, the following parameters were recorded by the attending physician at the time of the patient’s consultation: age, sex, body mass index, smoking history, vital signs (blood pressure, pulse rate, temperature, oxygen saturation) and the Alvarado score.^8^

### Outcomes

The reference standard for the diagnosis of acute appendicitis was based on a combination of clinical findings, imaging studies when deemed clinically appropriate, surgical findings and histopathological findings, and follow-up assessments. The diagnosis was adjudicated by the attending physicians involved in the patients’ clinical care, following standard clinical coding workflows. In non-surgical cases, the diagnosis was established by the attending physicians’ assessment, based on clinical evaluation and imaging reports if performed. The imaging criteria for diagnosing acute appendicitis were as follows:^14^,^16^

- US: Non-compressible appendix with a diameter≥6 mm, periappendiceal fluid or increased echogenicity of surrounding fat.
- CT: Enlarged appendix (≥6 mm in diameter), periappendiceal fat stranding or appendicolith.
- MRI: Enlarged appendix (≥6 mm in diameter), periappendiceal oedema or adjacent tissue inflammation

To reduce verification bias, all patients were re-evaluated by their attending physicians through follow-up phone calls or medical record reviews 7 days after the initial consultation to confirm their clinical status.

### Predictors

To quantitatively assess tongue coating, images were captured using a Nikon COOLPIX camera (resolution, 16.05 megapixels; focal-length range, 4.3–21.5 mm; aperture values, f/2.8 to f/8.0; Nikon, Tokyo, Japan). This camera was selected for its capacity to provide high-resolution images suitable for detailed analysis; images were captured before treatment initiation and saved for subsequent analysis. Photographs were obtained under prespecified conditions to maintain standardisation across the study.

The TCI, a reliable and valid method for the quantitative assessment of tongue coating, served as the index test.^12^ The TCI divides the tongue surface into nine sections, with the coating in each section being evaluated on a 3-point scale: a score of 0 indicates no coating, 1 indicates partial coating and 2 indicates thick coating. The scores for each section are summed, leading to a cumulative total score ranging from 0 to 18. The TCI was independently assessed by two raters who were not involved in the clinical care of the patients and who evaluated the images later, separate from the patients’ clinical visit. Both raters were blinded to the patients’ reference standard diagnoses to ensure that the TCI assessments were not influenced by knowledge of the final clinical outcome. For the analysis, the average TCI score from the two raters was used.

### Sample size estimation

Following the methodology described by Hajian-Tilaki for sample size calculation in diagnostic accuracy studies,^17^ we aimed for a sensitivity of 80% with a 5% margin of error and an estimated prevalence of 40% for acute appendicitis among the suspected cases. Given the clinical importance of high sensitivity in order to be able to rule out acute appendicitis (ie, high negative predictive value, NPV), we prioritised this metric among diagnostic performance parameters. Based on these criteria, the sample size calculation indicated that a minimum of 138 participants would be necessary to obtain reliable estimates of both sensitivity and specificity.

### Statistical analyses

Only complete cases with no missing data were included in the statistical analysis. Descriptive statistics of the patient clinical characteristics were calculated for the overall, appendicitis and non-appendicitis groups. Summary statistics were calculated after verifying data distribution. For continuous variables with a normal distribution, means and SD were computed; medians and IQRs were used for continuous variables without a normal distribution. For categorical variables, frequencies and percentages (%) were calculated. For comparisons between two groups, the Mann-Whitney U test was used for continuous variables without a normal distribution. For binary variables, Pearson’s χ^2^ test was applied.

The interexaminer reliability of the TCI was assessed using Bland-Altman plots. Diagnostic performance was evaluated for each examiner using the area under the curve (C-index AUC) analysis. Calibration curves were also generated to assess the consistency of diagnostic performance across examiners.

The discriminative ability of the overall Alvarado score and each score construct variable, as well as the TCI for diagnosing appendicitis was evaluated using the C-index AUC along with the corresponding 95% CI, through receiver operating characteristic (ROC) analysis.

A two-sided p value<0.05 was considered statistically significant. All statistical analyses were performed with EZR V.1.61 (Jichi Medical University, Saitama, Japan).

### Patient and public involvement

Patients or members of the public were not involved in the design, conduct or reporting of this clinical study.

## Results

### Participant enrolment flow and imaging studies

**Figure 1:**
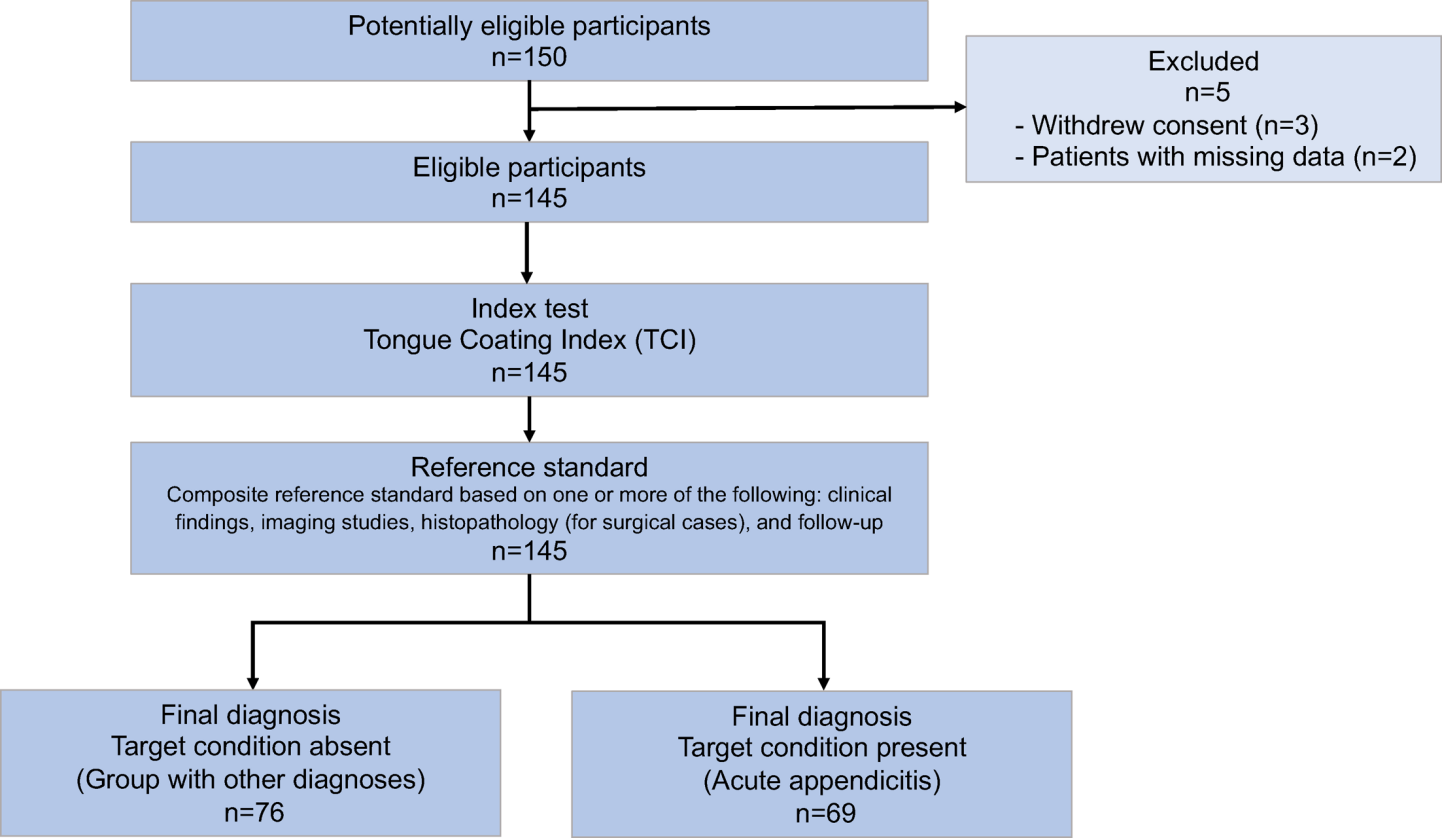
Standards for Reporting of Diagnostic Accuracy Studies flow diagram of participant enrolment and diagnostic evaluation process. This flow diagram illustrates the enrolment and classification of study participants over a 28-month period. A total of 150 patients were initially enrolled, with 5 excluded due to non-consent or missing data, resulting in 145 patients included in the analysis. These patients were categorised into two groups: those diagnosed with acute appendicitis (n=69) and those with other diagnoses (n=76). The reference standard was a composite reference standard, including clinical findings, imaging studies and follow-up for all patients. For surgical cases, histopathological findings were also included. The index test, Tongue Coating Index, was measured for all included patients using tongue images that were captured at the time of enrolment and evaluated at a later date.

Between 1 September 2018 and 31 December 2020, 150 patients were enrolled. After excluding three patients who did not consent and two patients with missing data, analyses were performed on 145 patients (figure 1).

### Patient characteristics

Patient characteristics and imaging results for the entire group and according to final diagnosis are shown in table 1. Among the 145 patients analysed, the median age was 43 years (IQR, 35–56) years, with 79 (55%) female patients. (table 1) Alongside the migration of pain (33% vs 59%), anorexia (63% vs 78%) and tenderness in the right lower quadrant (RLQ) (86% vs 96%), the median TCI was significantly higher in the acute appendicitis group (8 (IQR, 5–11)) than in the group with other diagnoses (6 (IQR, 4–9)) (p<0.05).

**Table 1:** Clinical characteristics of patients

	Overall (n=145)	Group with other diagnoses (n=76)	Acute appendicitis (n=69)	P value
Age, median (IQR), years	43 (35–56)	42 (29–50)	47 (35–70)	0.008
Female sex, no. (%)	79 (55)	43 (57)	36 (52)	0.590
Migration of pain, no. (%)	66 (46)	25 (33)	41 (59)	0.001
Anorexia, no. (%)	102 (70)	48 (63)	54 (78)	0.047
Nausea, no. (%)	66 (46)	35 (46)	31 (45)	0.890
Tenderness in the RLQ, no. (%)	131 (90)	65 (86)	66 (96)	0.039
Rebound pain, no. (%)	65 (45)	31 (41)	34 (49)	0.30
Elevated temperature, no. (%)	46 (32)	24 (32)	22 (32)	0.92
Leucocytosis, no. (%)	80 (56)	37 (50)	43 (62)	0.14
Alvarado score, median (IQR)	6 (4–7)	5 (4–7)	7 (5–8)	<0.001
TCI, median (IQR)	7 (4–10)	6 (4–9)	8 (5–11)	0.025
US performed, n (%)	132 (91)	70 (92)	62 (90)	
CT scans performed, n (%)	142 (97)	73 (96)	69 (100)	
MRI performed, n (%)	1 (0.7)	1 (1.3)	0 (0)	
No imaging, n (%)	2 (1.4)	2 (2.6)	0 (0)	

RLQ, right lower quadrant; TCI, Tongue Coating Index; US, ultrasonography.

**Figure 2:**
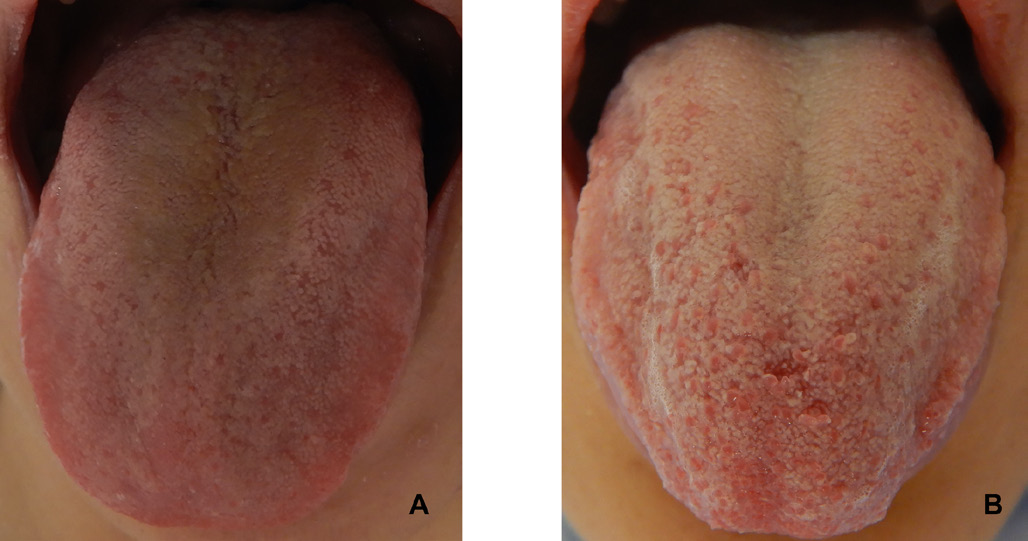
Comparative analysis of tongue coating thickness. (A) Patient with a diagnosis other than appendicitis, exhibiting a Tongue Coating Index (TCI) score of 1, indicating a thinner coating. (B) Patient diagnosed with acute appendicitis, characterised by a markedly thicker and unevenly distributed coating, with a TCI score of 13.5.

Figure 2 shows representative tongue images illustrating the difference in tongue coating between a patient with acute appendicitis (high TCI) and a patient with an alternative diagnosis (low TCI).

The Mann-Whitney U test was used for continuous variables with non-normal distributions, while Pearson’s χ^2^ test was used for categorical variables.

The most common other diagnoses were diverticulitis (25 cases, 17.2%) and acute enteritis (23 cases, 15.9%). Other conditions included caecal inflammation, pelvic inflammatory diseases (4 cases each, 2.8%), small bowel obstruction, acute pyelonephritis, ureteral stones (3 cases each, 2.1%), mesenteric lymphadenitis and functional gastrointestinal disorders (2 cases each, 1.4%). Less common diagnoses (1 case each, 0.7%) included acute cholecystitis, ovarian haemorrhage, pneumonia, menstrual pain and ruptured aneurysm.

### Discriminative ability of the TCI

The discriminative ability of the individual components of the Alvarado score, the overall score and the TCI for diagnosing acute appendicitis is summarised in table 2. Most C-index AUC values were within a similar range, with Migration of Pain, the composite Alvarado score and the TCI demonstrating statistically significant differences from 0.50. The TCI had a C-index AUC of 0.62 (95% CI, 0.53 to 0.71), which was comparable to individual variables of the Alvarado score, such as migration of pain to the RLQ (AUC 0.63 (95% CI, 0.55 to 0.71)), and to the composite Alvarado score (AUC 0.66 (95% CI, 0.57 t0 0.75)).

**Table 2:** Discriminative ability of components of the Alvarado score, total Alvarado score and TCI

Variables	C-index AUC (95% CI**)**	P value
Migration of pain	0.63 (0.55 to 0.71)	<0.001
Anorexia	0.58 (0.50 to 0.66)	NS
Nausea	0.49 (0.41 to 0.58)	NS
Tenderness in the RLQ	0.55 (0.50 to 0.60)	NS
Rebound pain	0.54 (0.46 to 0.62)	NS
Elevated temperature	0.50 (0.43 to 0.58)	NS
Leucocytosis	0.56 (0.48 to 0.64)	NS
Alvarado score	0.66 (0.57 to 0.75)	<0.001
TCI	0.62 (0.53 to 0.71)	0.003

The AUC was calculated using the ROC curve method. The p values were calculated using the z-test for the null hypothesis that AUC=0.5.

AUC, area under the curve; NS, not significant (p≥0.05); RLQ, right lower quadrant; ROC, receiver operating characteristic; TCI, Tongue Coating Index.

### Diagnostic accuracy of the Alvarado score and the TCI in predicting acute appendicitis

Table 3 summarises the diagnostic accuracy of the Alvarado score and TCI for predicting acute appendicitis. As there were no established thresholds for the TCI, the cut-off values were set exploratively in this study to provide potential thresholds depending on the clinical objective. For the goal of ruling out acute appendicitis, a TCI cut-off of 3 showed high sensitivity of 96% (95% CI, 88% to 98%) and NPV of 84% (95% CI, 62% to 94%), indicating its potential usefulness in identifying patients unlikely to have the condition. Conversely, a cut-off of 10 achieved higher specificity of 83% (95% CI, 73% to 90%) and PPV of 61% (95% CI, 44% to 75%), which could be helpful for confirming the diagnosis in patients with a higher pretest probability.

**Table 3:** Diagnostic accuracy of the TCI and the Alvarado score in predicting acute appendicitis

	TP	FN	FP	TN	Sensitivity (95% CI)	Specificity (95% CI)	PPV (95% CI)	NPV (95% CI)
Alvarado score ≥4	65	4	58	18	94% (86% to 98%)	24% (16% to 34%)	53% (44% to 62%)	82% (61% to 93%)
Alvarado score ≥5	60	9	47	29	87% (77% to 93%)	38% (28% to 49%)	56% (47% to 65%)	76% (61% to 87%)
Alvarado score ≥7	41	28	28	48	59% (48% to 70%)	63% (52% to 73%)	59% (48% to 70%)	63% (52% to 73%)
Alvarado score ≥9	10	59	6	70	15% (8% to 25%)	92% (84% to 96%)	63% (38% to 82%)	54% (48% to 63%)
TCI ≥3	66	3	60	16	96% (88% to 98%)	21% (13% to 32%)	52% (44% to 61%)	84% (62% to 94%)
TCI ≥10	20	49	13	63	29% (20% to 41%)	83% (73% to 90%)	61% (44% to 75%)	56% (47% to 65%)

FN, false negative; FP, false positive; NPV, negative predictive value; PPV, positive predictive value; TCI, Tongue Coating Index; TN, true negative; TP, true positive.

### Interexaminer reliability of the TCI

**Figure 3:**
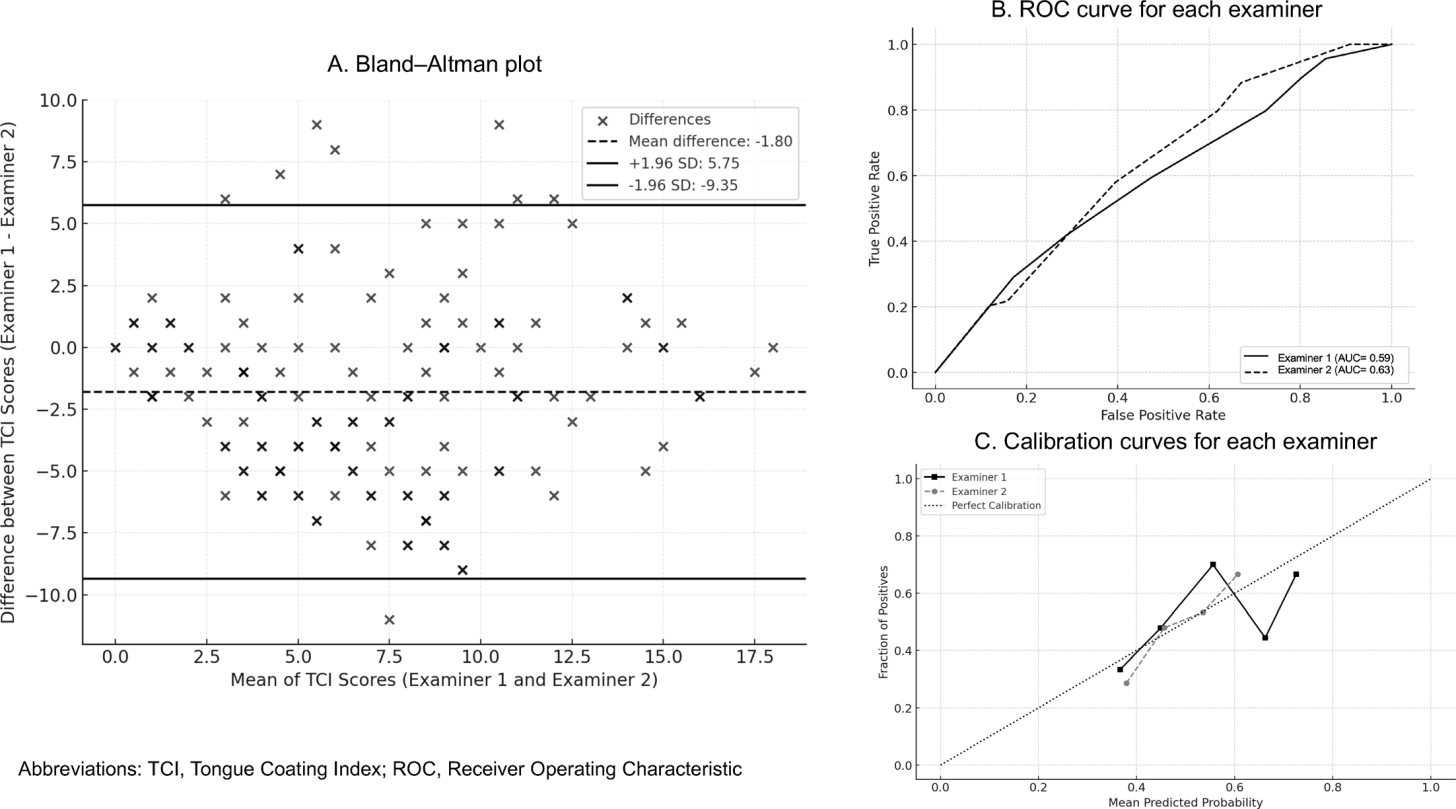
Interexaminer reliability and diagnostic performance of the TCI. (A) Bland-Altman plot for the TCI. The solid lines represent the limits of agreement (mean difference±1.96 SD) between the TCI scores assigned by two examiners, highlighting the range of variability in their assessments. (B)ROC curve for each examiner The C-index AUC for Examiner 1 was 0.59 (95% CI, 0.48 to 0.67), while Examiner 2 achieved a C-index AUC of 0.63 (95% CI, 0.53 to 0.71). Both values indicate moderate discriminative ability, with Examiner 2 performing slightly better in distinguishing between cases with and without acute appendicitis. (C) Calibration curves for each examiner The calibration curves illustrate the predicted probabilities versus observed outcomes for appendicitis for each examiner. Examiner 1 is represented by black squares with a solid line, while Examiner 2 is shown with grey circles and a dashed line. The dotted line represents perfect calibration, where predicted probabilities match observed outcomes exactly. The calibration curves illustrate the agreement between predicted probabilities and the actual occurrence of appendicitis for each examiner. AUC, area under the curve.

The agreement between the two examiners for TCI was evaluated using the Bland-Altman plot (figure 3A), which revealed variability between examiners in the TCI scores assigned. While differences in scoring were evident, a consistent relationship emerged: when one examiner assigned a higher TCI score, the other examiner tended to do the same, and similarly for lower scores.

Figure 3B presents the C-index AUC values, reflecting the correlation of the TCI scores assigned by each examiner with the clinical diagnosis of acute appendicitis. Examiner 1 achieved a C-index AUC of 0.59 (95% CI, 0.48 to 0.67), while Examiner 2 reached a slightly higher C-index AUC of 0.63 (95% CI, 0.53 to 0.71). These results indicate that both examiners showed moderate performance in scoring the TCI in a way that aligned with the presence or absence of acute appendicitis.

Calibration curves (figure 3C) were also generated to assess the accuracy of predicted probabilities relative to the observed outcomes. Both examiners demonstrated well-calibrated predictions, with their predicted probabilities closely matching the actual outcomes. This consistency reinforces the reliability of their diagnostic predictions and suggests that the TCI scoring, while variable between raters, maintained a generally accurate reflection of clinical outcomes.

## Discussion

This study suggests that the degree of tongue coating may be useful in the diagnosis of acute appendicitis, with performance comparable to the Alvarado score and its component of migration of pain. While tongue examination alone may not suffice for definitive diagnosis, its integration with other clinical signs could contribute to the development of new predictive models for acute appendicitis. Such models might enhance diagnostic accuracy, particularly in resource-limited settings where advanced imaging and laboratory tests are not readily available.

In previous studies, such as the systematic review by Kularatna *et al*,^7^ the diagnostic performance of the Alvarado score (using a cut‐off of 7) varied widely, with reported sensitivities ranging from 67.65% to 96.3% and specificities from 58.18% to 89.39%. This broad range suggests that the diagnostic performance of the Alvarado score is highly dependent on the underlying patient population. In studies reporting the upper limits of diagnostic accuracy, patients with more pronounced clinical features may have been enrolled, resulting in higher Alvarado scores. In contrast, our study enrolled patients with suspected appendicitis, many of whom exhibited intermediate Alvarado scores (4–7) that pose diagnostic challenges. Consequently, when using a cut-off of 7, our study demonstrated a sensitivity of 59% (95% CI, 48% to 70%) and a specificity of 63% (95% CI, 52% to 73%), which may have contributed to an overall lower AUC (0.62–0.68).

In our study, individual components of the Alvarado score demonstrated modest diagnostic performance. For instance, the migration of pain showed an AUC of 0.63 (95% CI, 0.55 to 0.71), anorexia 0.58 (95% CI, 0.50 to 0.66), and tenderness in the right lower quadrant 0.55 (95% CI, 0.50 to 0.60). These findings are in line with previous studies, which have similarly reported limited discriminative ability for individual clinical signs when used in isolation.^4^ This supports the notion that a composite score, such as the Alvarado score, is necessary to achieve improved diagnostic accuracy for acute appendicitis.

### Physiological implications of tongue coating

Tongue coating is thought to mirror the body’s physiological and pathological status, with studies indicating its potential association with gastritis, enteritis, diabetes, ischaemic heart diseases and cancer.^18^,^20^ Changes in the gut microbiota have been hypothesised to be a mechanism,^21 22^ which may also apply to appendicitis. Although Chinese medicine has documented appendicitis diagnoses using tongue examinations, the practical application of such studies has been limited because of the requirement of specialised equipment.^23 24^ Bailey and Clain also highlighted the importance of tongue coating in the diagnosis of acute appendicitis.^11^ Although their statement appears to be rooted in personal observations, our study supports the hypothesis.

This study possesses several strengths, notably its focus on a population with an Alvarado score primarily between 4 and 7, where the clinical diagnosis of acute appendicitis is particularly challenging. Additionally, the prospective collection of data with minimal missing values significantly bolsters the reliability of the findings. Recent advancements in diagnostic imaging, such as Point-of-Care Ultrasound (POCUS), have demonstrated high specificity and reduced radiation exposure compared with CT.^14^ However, POCUS requires operator expertise and equipment availability, which may limit its application in certain settings. In contrast, the TCI offers a simpler, non-technical alternative, potentially complementing tools like POCUS in resource-limited environments.

The study also enrolled patients based on clinical suspicion of acute appendicitis, a widely accepted approach in diagnostic accuracy research.^25^ This methodology reflects real-world clinical practice, where healthcare providers must assess patients suspected of appendicitis. By adopting this approach, we ensure the external validity of the study and mirror the typical patient population encountered in everyday clinical settings.

However, some limitations must be acknowledged. The study was conducted at a single institution, which may introduce spectrum bias and limit the generalisability of our findings.

Cases where acute appendicitis was not initially suspected or were treated as other conditions (eg, with antibiotics)—may not have been included. While these might represent a small proportion, clinically significant cases of acute appendicitis are likely to return for follow-up at our institution due to the healthcare structure in our region, minimising the risk of missing severe cases. Additionally, mild cases of appendicitis that could resolve spontaneously may also have been excluded, potentially introducing selection bias. However, such mild cases are generally not able to be included in most studies and are unlikely to influence the overall findings of the study.

While the TCI has been recognised as a reliable and valid indicator,^12^ our study observed variability between examiners, as shown in the Bland-Altman plots. However, the examiners demonstrated consistent scoring trends. The calibration curves and C-index AUC corroborate the overall accuracy and reliability of the TCI scores. Future research should focus on developing standardised and objective methods for measuring the TCI, potentially incorporating automated image analysis techniques. This could minimise interexaminer variability and enhance diagnostic precision.

Although histopathological assessment is traditionally the gold standard for diagnosing acute appendicitis, our study used a clinical diagnosis. Recent reports have highlighted a minimal difference in outcomes between surgical and conservative treatments for uncomplicated acute appendicitis,^26 27^ which has led to an increase in non-surgical management.

Implementing histopathology as the gold standard could reduce the number of definitively confirmed cases, thereby challenging the feasibility of the present study by limiting the sample size. Notably, all patients diagnosed with acute appendicitis in this study underwent CT, given its established reliability in diagnosing this condition.^4 28 29^

### Conclusion

The degree of tongue coating holds potential value as a useful, non-invasive diagnostic tool for acute appendicitis. Further research is necessary to validate these findings with external data and to explore the potential development of new decision aids for acute appendicitis, incorporating tongue examination as a component.

## Data Availability

Data are available upon reasonable request.

## References

[1] Humes DJ, Simpson J (2006). Acute appendicitis. BMJ.

[2] Bickell NA, Aufses AH, Rojas M (2006). How time affects the risk of rupture in appendicitis. J Am Coll Surg.

[3] Lewis SRR, Mahony PJ, Simpson J (2011). Appendicitis. BMJ.

[4] Andersson REB (2004). Meta-analysis of the clinical and laboratory diagnosis of appendicitis. Br J Surg.

[5] Eng KA, Abadeh A, Ligocki C (2018). Acute Appendicitis: A Meta-Analysis of the Diagnostic Accuracy of US, CT, and MRI as Second-Line Imaging Tests after an Initial US. Radiology.

[6] Fleming KA, Horton S, Wilson ML (2021). The Lancet Commission on diagnostics: transforming access to diagnostics. Lancet.

[7] Kularatna M, Lauti M, Haran C (2017). Clinical Prediction Rules for Appendicitis in Adults: Which Is Best?. World J Surg.

[8] Alvarado A (1986). A practical score for the early diagnosis of acute appendicitis. Ann Emerg Med.

[9] Howell JM, Eddy OL, Lukens TW (2010). Clinical policy: Critical issues in the evaluation and management of emergency department patients with suspected appendicitis. Ann Emerg Med.

[10] Malik AA, Wani NA (1998). Continuing diagnostic challenge of acute appendicitis: evaluation through modified Alvarado score. Aust N Z J Surg.

[11] Bailey H, Clain A (1954). Demonstrations of physical signs in clinical surgery.

[12] Shimizu T, Ueda T, Sakurai K (2007). New method for evaluation of tongue-coating status. J Oral Rehabil.

[13] Bossuyt PM, Reitsma JB, Bruns DE (2015). STARD 2015: an updated list of essential items for reporting diagnostic accuracy studies. BMJ.

[14] Boyle MJ, Lin-Martore M, Graglia S (2023). Point-of-care ultrasound in the assessment of appendicitis. Emerg Med J.

[15] Terasawa T, Blackmore CC, Bent S (2004). Systematic review: computed tomography and ultrasonography to detect acute appendicitis in adults and adolescents. Ann Intern Med.

[16] D’Souza N, Hicks G, Beable R (2021). Magnetic resonance imaging (MRI) for diagnosis of acute appendicitis. Cochrane Database Syst Rev.

[17] Hajian-Tilaki K (2014). Sample size estimation in diagnostic test studies of biomedical informatics. J Biomed Inform.

[18] Yin FG, Tian DL, Wang CH (1983). The relationship between fibergastroscopic picture and tongue inspection. J Tradit Chin Med.

[19] Jiang B, Liang X, Chen Y (2012). Integrating next-generation sequencing and traditional tongue diagnosis to determine tongue coating microbiome. Sci Rep.

[20] Han S, Chen Y, Hu J (2014). Tongue images and tongue coating microbiome in patients with colorectal cancer. Microb Pathog.

[21] Rauthan K, Joshi S, Kumar L (2023). Functional annotation of uncharacterized proteins from Fusobacterium nucleatum: identification of virulence factors. Genomics Inform.

[22] Aiyoshi T, Kakihara T, Watanabe E (2023). A comprehensive microbial analysis of pediatric patients with acute appendicitis. J Microbiol Immunol Infect.

[23] Pang B, Zhang D, Wang K (2005). Tongue image analysis for appendicitis diagnosis. Inf Sci (Ny).

[24] Zhang D, Pang B, Li N (2005). Computerized diagnosis from tongue appearance using quantitative feature classification. Am J Chin Med.

[25] O’Sullivan JW, Banerjee A, Heneghan C (2018). Verification bias. BMJ EBM.

[26] Salminen P, Paajanen H, Rautio T (2015). Antibiotic Therapy vs Appendectomy for Treatment of Uncomplicated Acute Appendicitis: The APPAC Randomized Clinical Trial. JAMA.

[27] Sallinen V, Akl EA, You JJ (2016). Meta-analysis of antibiotics versus appendicectomy for non-perforated acute appendicitis. Br J Surg.

[28] Hlibczuk V, Dattaro JA, Jin Z (2010). Diagnostic accuracy of noncontrast computed tomography for appendicitis in adults: a systematic review. Ann Emerg Med.

[29] Rud B, Vejborg TS, Rappeport ED (2019). Computed tomography for diagnosis of acute appendicitis in adults. Cochrane Database Syst Rev.

